# Genomic Sequence of the Prophage-Free Lactococcus lactis Strain IL6288

**DOI:** 10.1128/MRA.01545-18

**Published:** 2019-01-03

**Authors:** Alexander Bolotin, Anne Aucouturier, Alexei Sorokin, Elena Bidnenko

**Affiliations:** a MICALIS Institute, INRA, AgroParisTech, Université Paris-Saclay, Jouy-en-Josas, France; Georgia Institute of Technology

## Abstract

We report here the genome sequence of IL6288, a prophage-free derivative of Lactococcus lactis subsp. *lactis* strain IL1403, and confirm precise deletion of all prophages. Several single-nucleotide variations and an extra copy of the IS*981* element, apparently having a minor influence on cell physiology, were also detected in the IL6288 genome.

## Abstract (precis)

We report here the genome sequence of IL6288, a prophage-free derivative of Lactococcus lactis subsp. *lactis* strain IL1403, and confirm precise deletion of all prophages.

## ANNOUNCEMENT

Lactococcus lactis is an important lactic acid bacterium in industrial manufacturing of fermented dairy products (1). L. lactis subsp. *lactis* IL1403 is the first sequenced prototype lactococcal strain widely used for both fundamental and applied research (2). Construction and phenotypic characterization of its prophage-free derivative strain, named IL6288, were reported recently (3).

For sequencing, total bacterial DNA was extracted from 2 ml of overnight culture of IL1403 and IL6288 cells grown at 30°C in M17 medium supplemented with 0.5% glucose using the Wizard genomic DNA purification kit (Promega) as recommended by the supplier. Standard genomic libraries and sequencing reads were produced by Eurofins GATC Biotech, GmbH (Germany), using a HiSeq platform (Illumina). The complete genome sequences of strains IL1403 and IL6288 were attained using 2,698,237 and 3,152,569 paired-end 150-base-long reads, respectively. *De novo* assembly was done with SPAdes v.3.11.1 (4) and an iterative k-mer size-increasing protocol. Bandage v.0.8.1, Gap4 v.4.11.2-r, and SAMtools workflow (5–8) were used for repeat resolution, gap closure, and sequence correcting assisted by the available IL1403 sequence as a reference (9) (GenBank accession number NC_002662). The final sequences of the two strains are presented by a single contig each. SPAdes intermediate assemblies show coverage from 150× to 600× depending on the maximal k-mer used. The IL1403 reads were finally assembled into a 2,365,672-bp-long sequence, compared to the 2,365,589 bp of the reference, while the IL16288 reads produced a 2,208,218-bp-long contig. The genomes were automatically annotated with Prokka (10) v.1.12, which predicted 2,460 genes (2,379 protein-coding sequences [CDS], 19 rRNAs, and 61 tRNAs) for strain IL1403 and 2,218 genes (2,139 CDS, 19 rRNAs, and 59 tRNAs) for strain IL6288. Compared to the published genome of IL1403, done with MUMmer v.3.23 (11), the updated IL1403 sequence contains about 200 differences, which were mostly also found in the prophage-free IL6288 strain. The MUMmer-assisted genome alignment revealed, as expected, six additional regions (14.9, 36.9, 15.1, 35.5, 41.7, and 14.4 kb) in the IL1403 genome, corresponding to the deleted prophages. In the IL6288 chromosome, an extra copy of the IS*981* element integrated into an intergenic region was identified. Two of thirteen variations detected only in the IL6288 sequence were nonsynonymous substitutions in genes encoding manganese and maltose ABC transporters, while two others, in genes encoding chitinase and phosphate ABC transporter, appeared to be synonymous. The 5-bp deletion found in the gene encoding the arginine/ornitine antiporter resulted in a frameshift of translation over the C-terminal part of the protein. The remaining eight variations corresponded to CDS in repeated insertion sequence (IS) regions, and one was outside any CDS. In all likelihood, these variations should not influence the bacterial physiology to an extent similar to that of the deletion of resident prophages described recently (3).

### Data availability.

The complete genome sequences of IL1403 and IL6288 have been deposited in GenBank under the accession numbers CP033607 and CP033606, respectively. The IL1403 and IL6288 raw data were submitted to the NCBI Sequence Read Archive (SRA) as BioProject number PRJNA503975 under the SRA accession number SRP168462. The SRA accession numbers for the IL6288 and IL1403 runs are SRR8181920 and SRR8182677, respectively.
